# Endothelin Regulates *Porphyromonas gingivalis*-Induced Production of Inflammatory Cytokines

**DOI:** 10.1371/journal.pone.0167713

**Published:** 2016-12-28

**Authors:** Ga-Yeon Son, Eun-Jung Bak, Ji-Hye Kim, Dong Eun Lee, Si-Mook Kang, So Yun Lee, Lin Choi, Ji Su Sun, Seul Ki Kim, Wonse Park, Baek Il Kim, Yun-Jung Yoo, Inik Chang, Dong Min Shin

**Affiliations:** 1 Department of Oral Biology, Yonsei University College of Dentistry, Seoul, Republic of Korea; 2 BK21 PLUS Project, Yonsei University College of Dentistry, Seoul, Republic of Korea; 3 Department of Dental Hygiene, Jeonju Kijeon College, Jeonju, Republic of Korea; 4 Department of Preventive Dentistry and Public Oral Health, Yonsei University College of Dentistry, Seoul, Republic of Korea; 5 Department of Advanced General Dentistry, Yonsei University College of Dentistry, Seoul, Republic of Korea; Boston University Henry M Goldman School of Dental Medicine, UNITED STATES

## Abstract

Periodontitis is a very common oral inflammatory disease that results in the destruction of supporting connective and osseous tissues of the teeth. Although the exact etiology is still unclear, Gram-negative bacteria, especially *Porphyromonas gingivalis* in subgingival pockets are thought to be one of the major etiologic agents of periodontitis. Endothelin (ET) is a family of three 21-amino acid peptides, ET-1, -2, and -3, that activate G protein-coupled receptors, ET_A_ and ET_B_. Endothelin is involved in the occurrence and progression of various inflammatory diseases. Previous reports have shown that ET-1 and its receptors, ET_A_ and ET_B_ are expressed in the periodontal tissues and, that ET-1 levels in gingival crevicular fluid are increased in periodontitis patients. Moreover, *P*. *gingivalis* infection has been shown to induce the production of ET-1 along with other inflammatory cytokines. Despite these studies, however, the functional significance of endothelin in periodontitis is still largely unknown. In this study, we explored the cellular and molecular mechanisms of ET-1 action in periodontitis using human gingival epithelial cells (HGECs). ET-1 and ET_A_, but not ET_B_, were abundantly expressed in HGECs. Stimulation of HGECs with *P*. *gingivalis* or *P*. *gingivalis* lipopolysaccharide increased the expression of ET-1 and ET_A_ suggesting the activation of the endothelin signaling pathway. Production of inflammatory cytokines, IL-1β, TNFα, and IL-6, was significantly enhanced by exogenous ET-1 treatment, and this effect depended on the mitogen-activated protein kinases via intracellular Ca^2+^ increase, which resulted from the activation of the phospholipase C/inositol 1,4,5-trisphosphate pathway. The inhibition of the endothelin receptor-mediated signaling pathway with the dual receptor inhibitor, bosentan, partially ameliorated alveolar bone loss and immune cell infiltration. These results suggest that endothelin plays an important role in *P*. *gingivalis*-mediated periodontitis. Thus, endothelin antagonism may be a potential therapeutic approach for periodontitis treatment.

## Introduction

The endothelin (ET) system comprises three 21-amino acid peptides (ET-1, -2, and -3), two G protein-coupled receptors (ET_A_ and ET_B_), and activating peptidases, including endothelin converting enzymes (ECE-1 and -2). Endothelin, particularly ET-1, is implicated in many pathological states of diseases and is associated with the progress of chronic inflammation in a wide variety of organs through its ability to stimulate the production of inflammatory cytokines [1, 2].

Periodontitis is a chronic inflammatory disease of the periodontium, which leads to the destruction of both soft tissues and alveolar bone and the eventual exfoliation of the teeth. *Porphyromonas gingivalis* in subgingival pockets is a major etiologic pathogen for periodontitis acting through the stimulation of host cells to produce inflammatory cytokines, including interleukin (IL)-1β, IL-6, IL-8 and tumor necrosis factor (TNF) α[3]. Several studies have reported the potential involvement of ET-1 in periodontitis. Chen *et al*. suggested that endothelin expression is significantly increased in the gingiva of chronic periodontitis compared with the healthy one [4]. ET-1 peptide levels in gingival crevicular fluid and ET-1 mRNA expression in periodontal tissues from patients with periodontitis are higher than those from healthy subjects [5, 6]. Genotype analysis suggests that the interaction of the ET-1 gene with TNFβ and angiotensin-converting enzyme (ACE) may be involved in susceptibility to adult periodontitis [7]. Human gingival keratinocytes (HGKs) express endothelin mRNAs, and IL-1β and TNFα enhance ET-1 peptide production [5]. Conversely, ET-1 induces IL-1β expression in KB cells and HPdLF cells [8]. Recently, Liang *et al*. reported that ET-1 regulates pro-inflammatory cytokine production via the mitogen-activated protein kinase (MAPK) pathway in human periodontal ligament cells (HPDLs) [9]. In addition, ET-1 expression is strongly induced by *P*. *gingivalis* in KB cells and HEp-2 cells [6, 10].

The aforementioned findings imply the potential significance of endothelin in *P*. *gingivalis* infection and in subsequent inflammatory responses that lead to periodontitis. However, most of the studies discussed have been conducted with the epidermoid carcinoma cell line, KB, and the larynx carcinoma cell line, HEp-2 [6, 8, 10]. Unfortunately, these cell lines are contaminated with the cervical adenocarcinoma cell line, HeLa [11, 12]. Thus, it is necessary to verify the results of previous studies using physiological models such as primary human gingival epithelial cells (HGECs). Moreover, the functional role of *P*. *gingivalis*-induced endothelin and underlying mechanism of endothelin-mediated inflammatory cytokine production are still unclear. The effects of endothelin antagonism on the development and progression of periodontitis are also yet to be explored.

Therefore, in this study, we investigated the regulatory mechanisms of endothelin in *P*. *gingivalis*-mediated inflammatory response in HGECs. We further examined the effects of endothelin receptor inhibitors on alveolar bone loss and inflammation in an animal model of periodontitis.

## Materials and Methods

### Reagents

Keratinocyte Basal Medium-2 (KBM-2) was purchased from Lonza (Walkersville, MD, USA). Collagenase A and Dispase II were obtained from Roche (Mannheim, Germany). *P*. *gingivalis*, *P*. *gingivalis* LPS, *P*. *gingivalis* LPS-ulp, HKLM and Pam3 were purchased from Invivogen (San Diego, CA, USA). BQ123 and BQ788 were obtained from American Peptides Company (Sunnyvale, CA, USA). Bosentan was a product of MedChem Express (Monmouth Junction, NJ, USA). U73122, U73433, PD98059, SP600125, SB203580, 2-aminoethoxydiphenyl borate (2-APB), 1,2-Bis(2-aminophenoxy)ethane-N, N, N’, N’-tetraacetic acid tetrakis(acetoxymethyl ester) (BAPTA-AM) and *Escherichia coli* LPS were products of Sigma-Aldrich (St. Louis, MO, USA).

### Primary human gingival epithelial cells (HGECs) culture

HGECs were isolated from the gingival epithelium tissues resected during the extraction of healthy donor’s wisdom teeth as described previously [13]. In brief, the gingival tissues were separated from connective tissues after treatment with Collagenase A and Dispase II followed by Trypsin/EDTA. The isolated single-cell suspensions were maintained in KBM-2. HGECs within 3–4 passages were used for all experiments. All experimental protocol were reviewed and approved by the Research Ethics Committee of Yonsei University College of Dentistry and Dental Hospital. The written informed consent was obtained from all volunteers according to the requirements of the Institutional Review Board.

### Western blot analysis

Total proteins were extracted from the HGECs with radioimmunoprecipitation assay (RIPA) buffer (BioSolution Inc., Suwon, Korea) containing protease inhibitor cocktails (Roche Diagnostics, Risch-Rotkreuz, Switzerland). Total protein samples were run on the polyacrylamide gels using Mini-PROTEAN Tetra Cell Systems^®^ and then transferred to PVDF membranes in Mini Trans-Blot cell (BIO-RAD, Hercules, CA, USA). The membranes were incubated with antibodies against ET_A_, ET_B_, β-actin (Abcam, Cambridge, MA, USA), p-ERK, p-JNK, and p-p38 (Cell Signaling Technology, Danvers, MA, USA). The blots were labeled with horseradish peroxidase conjugated secondary antibody (Cell Signaling Technology). Specific complexes were visualized with an enhanced chemiluminescence (ECL) detection system (GE Healthcare, Little Chalfont, UK)

### Reverse transcription-polymerase chain reaction (RT-PCR)

Total RNA was extracted from HGECs using the Trizol reagent (Invitrogen, Waltham, MA, USA), and then cDNA synthesis was performed using AccuPower^®^ RT PreMix (BIONEER, Daejeon, Korea) for conventional PCR and Superscript III (Invitrogen) for quantitative PCR. For conventional PCR, cDNAs were amplified with EmeraldAmp GT PCR master mix (Takara Bio Inc., Shiga, Japan) and the products were visualized on a 1.5% agarose gels. SensiFAST SYBR Hi-ROX Kit (Bioline, Taunton, MA, USA) was used for quantitative PCR. The sequences of primers used are listed in S1 Table.

### Inflammatory cytokine measurement

Amount of secreted inflammatory cytokines was measured with supernatant from HGECs using Qunatikine enzyme-linked immunosorbent assay (ELISA) kit for human IL-1β, TNFα and IL-6 (Minneapolis, MN, USA). The epithelialcell viability was >90% and the value were normalized with cell number.

### *P*. *gingivalis* infection

*P*. *gingivalis* 33277 were grown anaerobically (85% N_2_, 10% H_2_, and 5% CO_2_) in brain heart infusion medium with hemin and vitamin K. The bacteria, at multiplicities of infection (MOI) of 100, were incubated with HGECs in antibiotic-free HGECs culture medium. After washing with phosphate-buffered saline, the remaining external bacteria were killed with metronidazole and gentamicin. No effects of *P*. *gingivalis* (100 MOI) infection on the viability and growth of cells were presented in Supporting Information (S1 Fig). *P*. *gingivalis* LPS standard (Pg LPS) is produced by a standard LPS preparation. It exhibits TLR2 activity due to a contaminant lipoprotein and also activates cells through TLR4 [14–16]. *P*. *gingivalis* LPS ultrapure (Pg LPS-ulp) underwent enzymatic treatment to remove lipoproteins and thus only activates TLR4. Endotoxin-free water utilized to dissolve both Pg LPS and Pg LPS was used as the solvent control in all the experiments.

### Cell viability and proliferation assay

After *P*. *gingivalis* infection, apoptotic death was examined by staining cells with an Annexin V-fluorescein isothiocyanate (FITC)/7-amino-actinomycin D (7-AAD) (BD Biosciences, San Diego, CA, USA) as described by the manufacturer and analyzing with LSR II (BD Biosciences). Both early (Annexin V-positive/7-AAD-negative) and late (Annexin V-positive/7-AAD-positive) apoptotic cells were included in cell death determinations. Cell proliferation was determined by adding MTS (3-(4,5-dimethylthiazol-2-yl)-5-(3-carboxymethoxyphenyl)-2-(4-sulfophenyl)-2H-tetrazolium)-based CellTiter 96 AQ_ueous_ One Solution Reagent (Promega, Madison, WI, USA) and measuring the absorbance at 490 nm on SPECTRA MAX 190 plate reader (Molecular Devices, Sunnyvale, CA, USA).

### Measurement of intracellular Ca^2+^ concentration ([Ca^2+^]_i_)

Cultured HGECs on collagen-coated cover glasses were loaded with Fura-2/AM. Changes in [Ca^2+^]_i_ were measured by means of fura-2 fluorescence with excitation wavelengths of 340 and 380 nm, respectively, and an emission wavelength of 510 nm. The emitted fluorescence was obtained at 2 s intervals and analyzed with a MetaFluor system (Molecular Devices).

### Experimental design of animal study

After 1-week acclimation, 6-week old male C57BL/6 mice (Orient-Bio Inc., Seongnam, Korea) were divided into the following four groups: control with vehicle (Con+Veh), control with bosentan (Con+Bos), periodontitis with vehicle (Perio+Veh), and periodontitis with bosentan (Perio+Bos) (n = 6 to 10 in each group at each time point). For ligature-induced periodontitis, the mice were anesthetized intraperitoneally with a 1:2 mixture of Zoletil^®^ (30 mg/kg; Virbac, Carros, France) and Rompun^®^ (10 mg/kg; Bayer Korea, Ansan, Korea). Dental floss was placed around the cervix of the bilateral first molar and knotted mesially to induce periodontitis [17]. Bosentan was prepared fresh every day as a suspension in 5% gum arabic (Sigma-Aldrich). Vehicle (5% gum arabic) and bosentan (30 mg/kg) administrated daily via gastric gavage. On day 3 or 9 after ligature, mice were sacrificed by CO_2_ inhalation and the mandibles were dissected and analyzed histologically. During the experimental period, the location of dental floss and gross appearance of mice were observed daily. Animal protocols were approved by the Institutional Animal Care and Use Committee of the Yonsei University Health System (2015–0146).

### Histologic examination

The dissected mandibles were fixed in 10% neutral buffered formalin overnight and decalcified in 5% nitric acid for 1 week. The paraffin-embedded sections were selected on clear appearance of the dental pulp of the mesial and distal roots of the first molars and were stained with hematoxylin and eosin [17]. For estimation of alveolar bone loss, the distance from the alveolar bone crest (ABC) to the cementoenamel junction (CEJ) in the distal area and the percentage of periodontal ligament area to the region of interest (ROI) in the furcation area were evaluated under ×100 magnification (Olympus CKX41, Tokyo, Japan) and ImagePro software (Media Cybernetics, Silver Spring, MD, USA). The height of the ROI was 0.8 mm from the top of the furcation. For evaluation of inflammation, the number of polymorphonuclear and mononuclear cells were counted in two standardized sites (0.1 × 0.1 mm) located under the junctional epithelium of the distal area of the first molar (×400 magnification). The evaluation was conducted by an independent examiner in a blind manner.

### Statistical analyses

Values are presented as the mean ± standard error of mean (SEM) based on results obtained from at least three independent experiments. All statistical analyses were carried out using GraphPad PRISM Software. Two-tailed unpaired Student’s *t*-test was used for comparisons between two groups. One-way analysis of variance (ANOVA) followed by Tukey post hoc test was used to identify any significant differences of the distance from CEJ to ABC, and the number of inflammatory cells after bosentan treatment. A *P* value of <0.05 was regarded as statistically significant.

## Results

### *P*. *gingivalis* induces ET-1 production in HGECs

**Fig 1:**
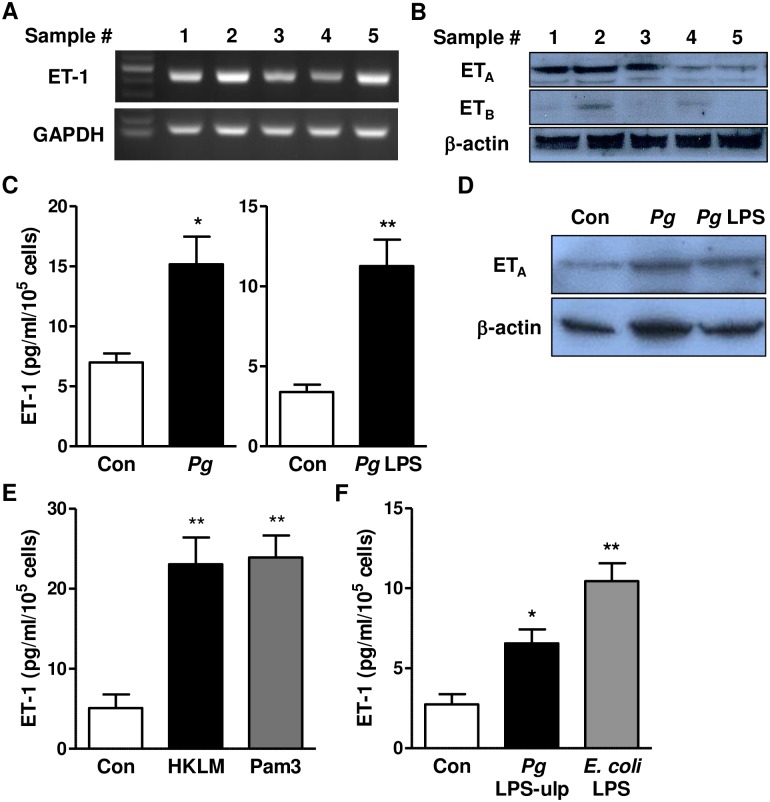
Endothelin expression in human gingival epithelial cells (HGECs) following *P*. *gingivalis* infection. (A and B) Expression of ET-1 mRNA (A), and ET_A_ and ET_B_ protein (B) in HGECs was analyzed by RT-PCR and western blot, respectively. (C and D) Induction of ET-1 peptide and ET_A_ protein by *P*. *gingivalis* and *P*. *gingivalis* LPS. After 18h infection with *P*. *gingivalis* (100 MOI) and 24h treatment with *P*. *gingivalis* LPS (1 μg/ml), ET-1 peptide and ET_A_ protein levels were analyzed by ELISA and western blot, respectively. ET-1 levels were measured in culture supernatants from five samples (sample #1–5). A representative blot from sample #1 is shown. *P<0.05; **P<0.01. (E and F) Involvement of TLR in ET-1 production. After 24h treatment with HKLM (10^8^ cells/ml), or Pam3 (50 ng/ml) (E), or Pg LPS-ulp (1 μg/ml) or *E*. *coli* LPS (50 ng/ml) (F), ET-1 production was evaluated by ELISA in culture supernatants from five samples (sample #1–5). *P<0.05; **P<0.01.

To determine the role of endothelin in HGECs, we first examined the expression of ET-1 and its receptors, ET_A_ and ET_B_. ET-1 was expressed in all examined 25 samples and ET_A_ was dominantly present in HGECs (Fig 1A and 1B and S2 Fig). Next, we investigated the effect of *P*. *gingivalis* on the levels of ET-1 and ET_A_. Both *P*. *gingivalis* and *P*. *gingivalis* LPS (*Pg* LPS) up-regulated the expression of ET-1 and ET_A_, indicating the activation of the ET-1/ET_A_ signaling pathway by *P*. *gingivalis* (Fig 1C and 1D and S3 Fig). Although *Pg* LPS is recognized predominantly by TLR4 [18], it also activates TLR2 due to a lipoprotein contaminant [16]. To examine whether TLR activation is involved in *P*. *gingivalis*-mediated ET-1 production, we treated HGECs with TLR2 agonists (HKLM and Pam3) or a TLR4 agonist (*Pg* LPS-ulp and *E*. *coli* LPS). As shown in Fig 1E and 1F, ET-1 was significantly increased by the agonists of TLR2 and TLR4 implying the involvement of TLR activation for the ET-1 production.

### ET-1 mediates *P*. *gingivalis*-induced production of inflammatory cytokines

**Fig 2:**
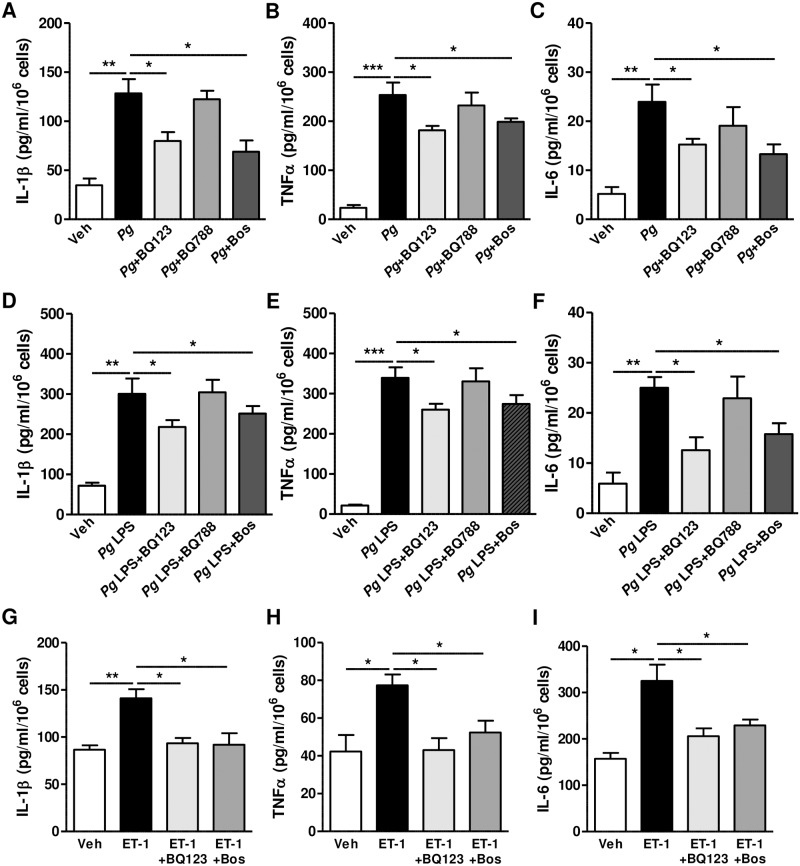
Effect of endothelin inhibitors on the production of inflammatory cytokines in HGECs. (A to C) Reduced production of IL-1β (A), TNFα (B), and IL-6 (C) after treatment with endothelin receptor antagonists and *P*. *gingivalis*. After treatment with BQ123 (100 nM), BQ788 (100 nM), or bosentan (100 nM) for 1h, cells were infected with *P*. *gingivalis* (100 MOI) for 24h. (D to F) Reduced production of IL-1β (D), TNFα (E), and IL-6 (F) after treatment with endothelin receptor antagonists and *P*. *gingivalis* LPS (1 μg/ml). After treatment with BQ123 (100 nM), BQ788 (100 nM), or bosentan (100 nM) for 1h, cells were treated with *P*. *gingivalis* LPS (1 μg/ml) for 24h. (G to I) Reduced production of IL-1β (G), TNFα (H), and IL-6 (I) after treatment with endothelin receptor antagonists and ET-1. After pre-treatment with BQ123 (100 nM), BQ788 (100 nM), and bosentan (100 nM) for 1h, cells were treated with ET-1 (100 nM) for 24h. Secretion of cytokines or ET-1 was examined by ELISA in the culture supernatants from five samples (sample #1–5). *P<0.05; **P<0.01; ***P<0.001.

To determine the role of ET-1 which is induced by *P*. *gingivalis* infection, we assessed the production of inflammatory cytokines after treatment with inhibitors of endothelin receptors. *P*. *gingivalis* induces the production of IL-1β, TNFα, and IL-6, and this induction was significantly reduced by BQ123, an ET_A_ blocker. In agreement with our results showing negligible expression of ET_B_ in HGECs (Fig 1B), inhibition of ET_B_ by BQ788 had no effect on the production of inflammatory cytokines. Bosentan, a dual inhibitor of ET_A_ and ET_B_ prevented the secretion of inflammatory cytokines, which could be the result of ET_A_ inhibition (Fig 2A to 2C). *Pg* LPS also induced the production of IL-1β, TNFα, and IL-6, and this was attenuated by treatment with BQ123 or bosentan, but not with BQ788 (Fig 2D to 2F). Next, we examined whether the ET_A_ signaling pathway, activated by ET-1, directly affects the production of inflammatory cytokines in HGECs. As shown in Fig 2G to 2I, ET-1 directly induced the secretion of IL-1β, TNFα, and IL-6 in HGECs, and inhibition of ET_A_ with BQ123 or bosentan resulted in a reversal of the influence of ET-1 on cytokine production.

### ET-1 stimulates the production of inflammatory cytokines via Ca^2+^-activated signaling pathway

**Fig 3:**
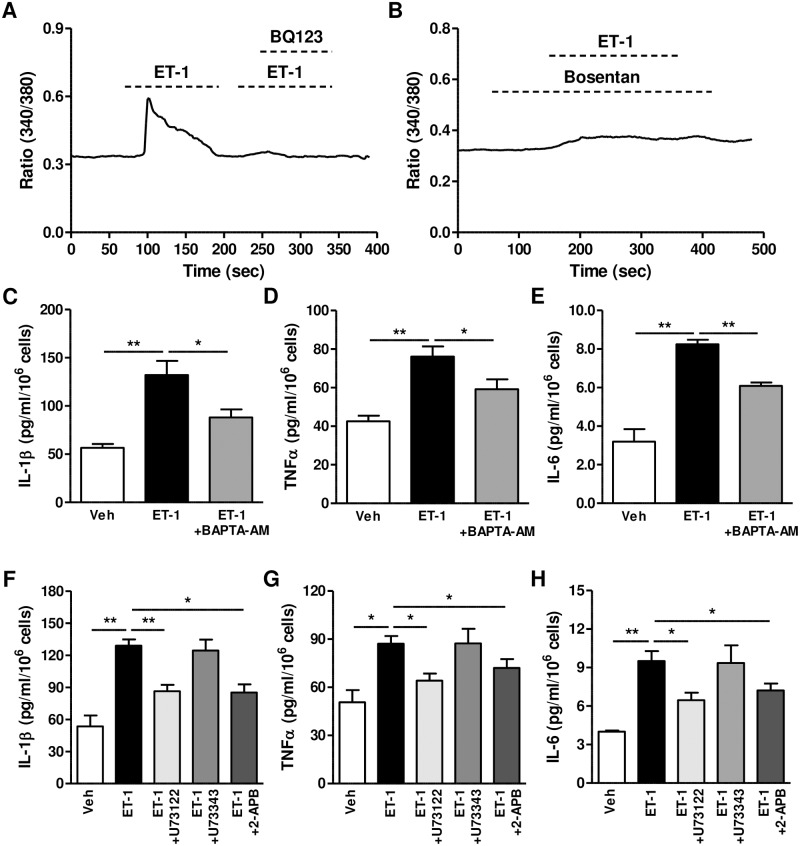
Involvement of the Ca^2+^/PLC/IP_3_ pathway in endothelin-mediated inflammatory cytokine production. (A and B) ET-1-induced increase in intracellular Ca^2+^ concentration [Ca^2+^]_i_. The fura-2/AM- stained cells were treated with ET-1 and BQ123 (100 nM) (A) or bosentan (100 nM) (B). Change of [Ca^2+^]_i_ was measured through fluorescence intensity at excitation wavelengths of 340 and 380 nm. (C to E) Effect of BAPTA-AM on ET-1-mediated production of inflammatory cytokines. After pre-treatment with BAPTA-AM (5 μM) for 20 min, HGECs were treated with ET-1 (100 nM) for 24h and production of IL-1β (F), TNFα (G), and IL-6 (H) was measured by ELISA in culture supernatants from five samples (sample #1–5). *P<0.05; **P<0.01. (F to H) Involvement of the PLC/IP_3_ pathway in the ET-1-induced production of inflammatory cytokines. After pre-treatment with U73122 (10 μM), U73342 (10 μM), or 2-APB (75 μM) for 30 min, HGECs were treated with ET-1 (100 nM) for 24h and production of IL-1β (C), TNFα (D), and IL-6 (E) was measured by ELISA in culture supernatants from five samples (sample #1–5). *P<0.05; **P<0.01.

**Fig 4:**
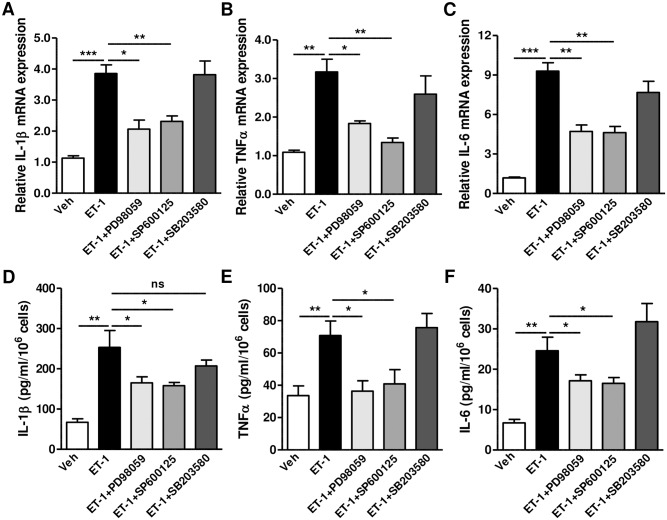
Involvement of MAPK in endothelin-mediated inflammatory cytokine production. (A to C) Effect of MAPK inhibitors on the ET-1-mediated mRNA expression of inflammatory cytokines. After pre-treatment with PD98059 (10 μM), SP600125 (10 μM), and SB203580 (10 μM) for 20 min, HGECs were treated with ET-1 (100 nM) for 24h and mRNA expression of IL-1β (F), TNFα (G), and IL-6 (H) was measured by quantitative RT-PCR. *P<0.05; **P<0.01; **P<0.001. (D to F) Effect of MAPK inhibitors on the ET-1-mediated production of inflammatory cytokines. After pre-treatment with PD98059 (10 μM), SP600125 (10 μM) and SB203580 (10 μM) for 20 min, HGECs were treated with ET-1 (100 nM) for 24h and production of IL-1β (F), TNFα (G), and IL-6 (H) was measured by ELISA in culture supernatants from five samples (sample #1–5). *P<0.05; **P<0.01; ns: non-significant.

Because ET_A_ is a G_q_ protein-coupled receptor, we examined whether the phospholipase C/inositol 1, 4, 5-trisphosphate (PLC/IP_3_)-mediated intracellular Ca^2+^ concentration ([Ca^2+^]_i_) increase is involved in the production of inflammatory cytokines. ET-1 stimulated the increase in [Ca^2+^]_i_ in HGECs, and this was prevented by the ET_A_ receptor blockers, BQ123 and bosentan (Fig 3A and 3B). To directly examine whether the increase in [Ca^2+^]_i_ can influence the secretion of inflammatory cytokines, we exposed HGECs to ET-1, along with the intracellular Ca^2+^-selective chelator, BAPTA-AM. The ET-1-induced secretion of inflammatory cytokines was markedly repressed by BAPTA-AM (Fig 3C to 3E). In addition, to determine the role of the PLC/IP_3_ pathway in ET-1-induced inflammatory cytokine production, we treated HGECs with U73122, a specific inhibitor of PLC, or its inactive analog, U73343, and the IP_3_ receptor blocker, 2-APB. As shown in Fig 3F to 3H, U73122, but not U73343, suppressed ET-1-induced production of inflammatory cytokines. 2-APB also inhibited cytokine expression. Because MAPKs, which have been shown to be involved in ET-1-mediated inflammatory cytokine production in HPDLs, are influenced by Ca^2+^ signaling [9, 19], we explored the involvement of MAPKs in HGECs. As shown in S4 Fig, JNK and ERK1/2 but not p38 were activated by *Pg*, *Pg* LPS and ET-1. In addition, induction of IL-1β, TNFα, and IL-6 by ET-1 was significantly blocked by inhibition of JNK (SP600125) and ERK1/2 (PD98059) but inhibition of p38 (SB203580) had little effect on ET-1-induced mRNA (Fig 4A to 4C) and protein (Fig 4D to 4F) expression of cytokine.

### Inhibition of endothelin signaling partially ameliorates the progression of periodontitis

**Fig 5:**
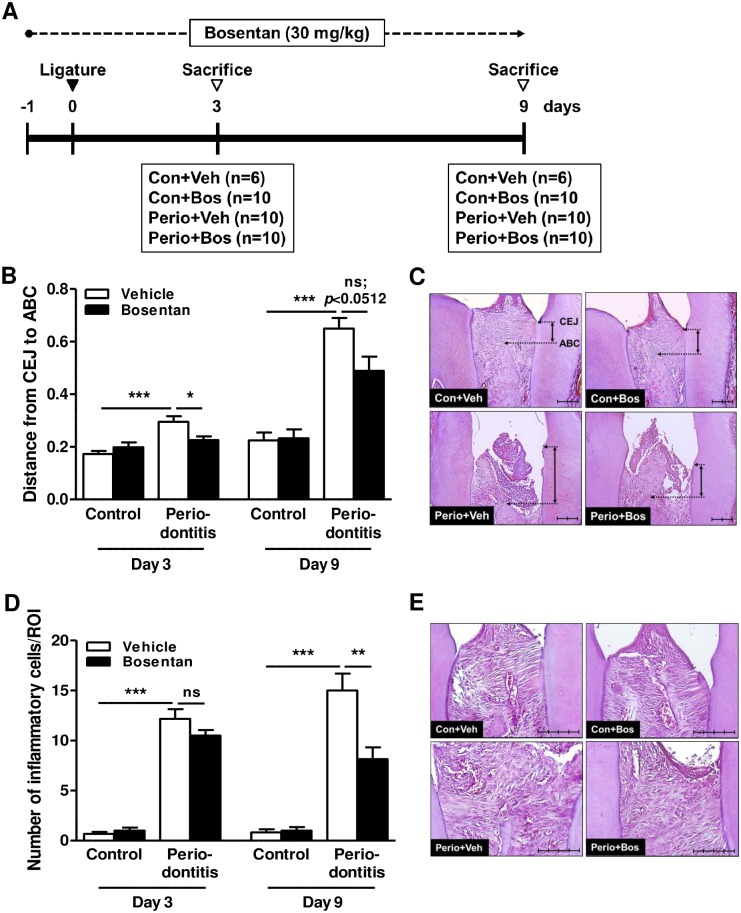
Effect of endothelin inhibition on ligature-induced periodontitis. (A) Experimental design and time schedule. Ligature was placed on day 0, and bosentan was treated 1 day before ligature ligation (-1 day). On days 3 and 9 after ligature ligation, the mandibles were dissected and histomorphometric and immunohistochemical analyses were performed. (B and C) Effect of endothelin inhibition on alveolar bone loss on days 3 and 9. ABC-CEJ distances measured by histomorphometric analysis (B) are shown along with representative image (C). *P<0.05; ***P<0.001; ns: non-significant. (D and E) Effect of endothelin inhibition on the number of inflammatory cells in the gingiva on days 3 and 9. Number of inflammatory cells (D) measured by immunohistochemical analysis are shown along with the representative image (E). **P<0.01; ***P<0.001. Scale bar = 100 μm; original magnification ×20.

Bosentan prevented the secretion of inflammatory cytokines (Fig 2G to 2I). Thus, we evaluated the beneficial effects of bosentan on the mice with ligature-induced periodontitis (Fig 5A). Morphometric analysis demonstrated that the distance from the cementoenamel junction (CEJ) to the alveolar bone crest (ABC) in the control group treated with vehicle was shorter than that in the periodontitis group throughout the experimental period. In the periodontitis group, at early time point, the mice that received bosentan exhibited less severe bone loss than those that received vehicle (day 3; CEJ-ABC distance of 0.30 ± 0.02 mm and 0.22 ± 0.01 mm, respectively). Despite the statistical insignificance, the mice that received bosentan tended to display less severe bone loss at later time point than those that received vehicle (day 9; Fig 5B). In accordance with the morphometric results, the histological images revealed that the bone crest and junctional epithelium remained normal in the control group. However, in the periodontitis group, alveolar bone resorption was extensive, and the junctional epithelium was elongated. These effects were reduced in the bosentan treatment (Fig 5C). As illustrated in Fig 5D, an increased number of inflammatory cells was detected in the periodontitis group compared with the control group. However, at later time point, bosentan treatment significantly reduced the number of inflammatory cells (day 9; Fig 5D and 5E).

## Discussion

*P*. *gingivalis* has been regarded as one of the major etiologic agents in chronic periodontitis. Interestingly, it produces endopeptidase PgPepO, which has a significant homology with endothelin-converting enzyme (ECE)-1 [20]. Although further investigation is needed, this points to the possibility that the activation of PgPepO after *P*. *gingivalis* infection may contribute to ET-1 production in host cells. A previous report shows that a PgPepO-deficient strain has a lower capability of invasion compared to wild type strains [21]. This implies that *P*. *gingivalis* may use endothelin for the bacterial invasion process. In addition, ET-1 may induce host inflammation of periodontal tissues in response to *P*. *gingivalis* infection.

ET-1 is induced by *P*. *gingivalis* in KB and HEp-2 cell lines, which were unfortunately contaminated with HeLa cells [11, 12], but little information has been revealed on the underlying molecular mechanism. Therefore, ET-1 induction should be verified and the underlying mechanisms need to be revealed in an appropriate system reflecting oral environment. In this study, we found the expression of ET-1 and ET_A_ in HGECs and both TLR2 and TLR4 are involved in *P*. *gingivalis*-mediated ET-1 production. It would be of interest to examine whether PgPepO, through ET-1 production, evokes an immune response via the TLR signaling pathway in HGECs as has been shown in immune system activation that results from the interaction of *Streptococcus pneumonia* endopeptidase O with TLRs [22].

Endothelin signaling has been shown to directly activate the immune response and the infiltration of immune cells into the site of inflammation [23]. *P*. *gingivalis* up-regulates the expression of ET-1 along with IL-1β, IL-8, and intercellular adhesion molecule (ICAM-1), in HEp-2 cells [6]. Rikimaru *et al*. showed that ET-1 stimulates IL-1β production in KB and PDL cells [8]. Because the progression of periodontitis is closely related with the expression of inflammatory cytokines such as IL-1β, -6, -8 and TNFα in gingival tissues [24, 25], ET-1 could play important roles in the initiation and progression of the disease through the regulation of cytokine expression. The signaling pathways involved in ET-1-mediated cytokine production via MAPKs have been investigated in HPDLs [9]. Our study indicates that ET-1 activates ERK1/2 and JNK, but not p38 in HGECs (Fig 4). Additionally, we report the novel finding that the PLC/IP_3_ pathway-mediated increase in [Ca^2+^]_i_ plays a critical role in ET-1 function. For these experiments, we have used chemical inhibitors to determine the ET-1-mediated signal transduction. However, genetic knockdown approaches using si- or shRNAs will be required to exclude the potential off-target effects of the chemicals and to entirely confirm the conclusions. Several inflammatory cytokines, such as IL-1β, -6, and -8, are known to up-regulate ET-1 production [26]. Moreover, Fujioka *et al*. showed that ET-1 mRNA expression and peptide production are increased by IL-1β and TNFα in human gingival keratinocytes [5]. Thus, the reciprocal interaction between ET-1 and inflammatory cytokines may contribute to the long-term progression of periodontitis after *P*. *gingivalis* infection. ET-1 and inflammatory cytokines may be interdependent and may establish an inflammatory loop, which can contribute to the incidence and progression of chronic periodontitis.

Activation of inflammatory cytokines results in alveolar bone loss by inducing osteoclast formation [27]. It has been suggested that ET-1 is involved in orthodontic tooth movement probably by enhancing bone resorption [28]. In addition to an indirect participation in periodontitis via the induction of inflammatory cytokines, ET-1 may directly aggravate the disease through the enhancement of osteoclast formation, eventually leading to alveolar bone loss.

Inhibitors of endothelin receptors prevent the development of inflammation-related diseases such as colitis, airway inflammation, and collagen-induced arthritis [29–31]. In our study, bosentan alleviates alveolar bone loss and infiltration of inflammatory cells in an animal model of ligature-induced periodontitis (Fig 5). However, bosentan was not fully effective during the entire experimental period. Bosentan treatment was statistically potent only at the early stages of alveolar bone loss during the course of the disease, and its effect was diminished at later time points (Fig 5B). In periodontitis, receptor activator of nuclear factor-kB ligand (RANKL) is a critical factor for alveolar bone loss along with pro-inflammatory cytokines [32, 33]. Interestingly, ET-1 did not affect RANKL secretion in HGECs (S5 Fig). Thus, it is likely that bosentan was able to prevent pro-inflammatory cytokine-mediated bone loss at earlier time point but was unable to inhibit RANKL-induced bone destruction later. It would be interesting to know whether the secretion of pro-inflammatory cytokines and of RNAKL occurs sequentially. Our results suggest that long-term treatment of bosentan or co-treatment with a RANKL inhibitor such as osteoprotegerin (OPG), might be more effective for the prevention of bone loss.

In the present study, we reported that the induction of ET-1 and ET_A_ by *P*. *gingivalis* plays an important role in the production of inflammatory cytokines in HGECs. In addition, we demonstrated that MAPK activation via the increase in intracellular Ca^2+^ that results from the activation of the PLC/IP_3_ is the underlying mechanism of ET-1 action. Finally, we showed that inhibition of ET-1/ET_A_ with bosentan partially ameliorated alveolar bone loss and immune cell infiltration suggesting the potential therapeutic approach of endothelin antagonism for periodontitis.

## Supporting Information

S1 Table List of primer sequences used in the study. (TIF)

S1 Fig No effect of P. gingivalis infection on the viability and growth of HGECs. (A and B) After 18h infection with *P*. *gingivalis* (100 MOI) apoptotic cell death was determined by flow cytometric analysis using double staining with Annexin V-FITC and 7-AAD (A) and cell growth was examined by MTS assay (B).
(PPTX)

S2 Fig Additional analysis of endothelin expression in HGECs. RNA was extracted from human gingival epithelial cells and mRNA expression of ET-1, ET_A_ and ET_B_ in HGECs was analyzed by RT-PCR. GAPDH was used as loading control.
(PPTX)

S3 Fig Induction of mRNAs of ET-1 and ET_A_ by *Pg* or *Pg* LPS. ET-1 (A and B) or ET_A_ (C and D) mRNA expression was examined by quantitative RT-PCR after *Pg* (100 MOI; A and C) infection for 18h or *Pg* LPS (1 μg/ml; B and D) treatment for 24h. *P<0.05; **P<0.01; **P<0.001.
(PPTX)

S4 Fig Activation of MAPKs by *Pg*, *Pg* LPS or ET-1. Phosphorylation of JNK, ERK and p38 was examined by Western blot with phosphorylation-specific antibodies. β-actin was used as a loading control.
(PPTX)

S5 Fig No effect of ET-1 on the production of RANKL and OPG in HGECs. (A and B) ET-1 (100 nM) was treated at the indicated times, and the secretion of RANKL (A) and OPG (B) was examined by ELISA in culture supernatants from five samples (sample #1–5).
(PPTX)
