# Supplementary figures and images for: Endothelin Regulates Porphyromonas gingivalis-Induced Production of Inflammatory Cytokines
Source: PLoS One. 2016 Dec 28;11(12):e0167713. doi: 10.1371/journal.pone.0167713 (PMC5193354; doi:10.1371/journal.pone.0167713)

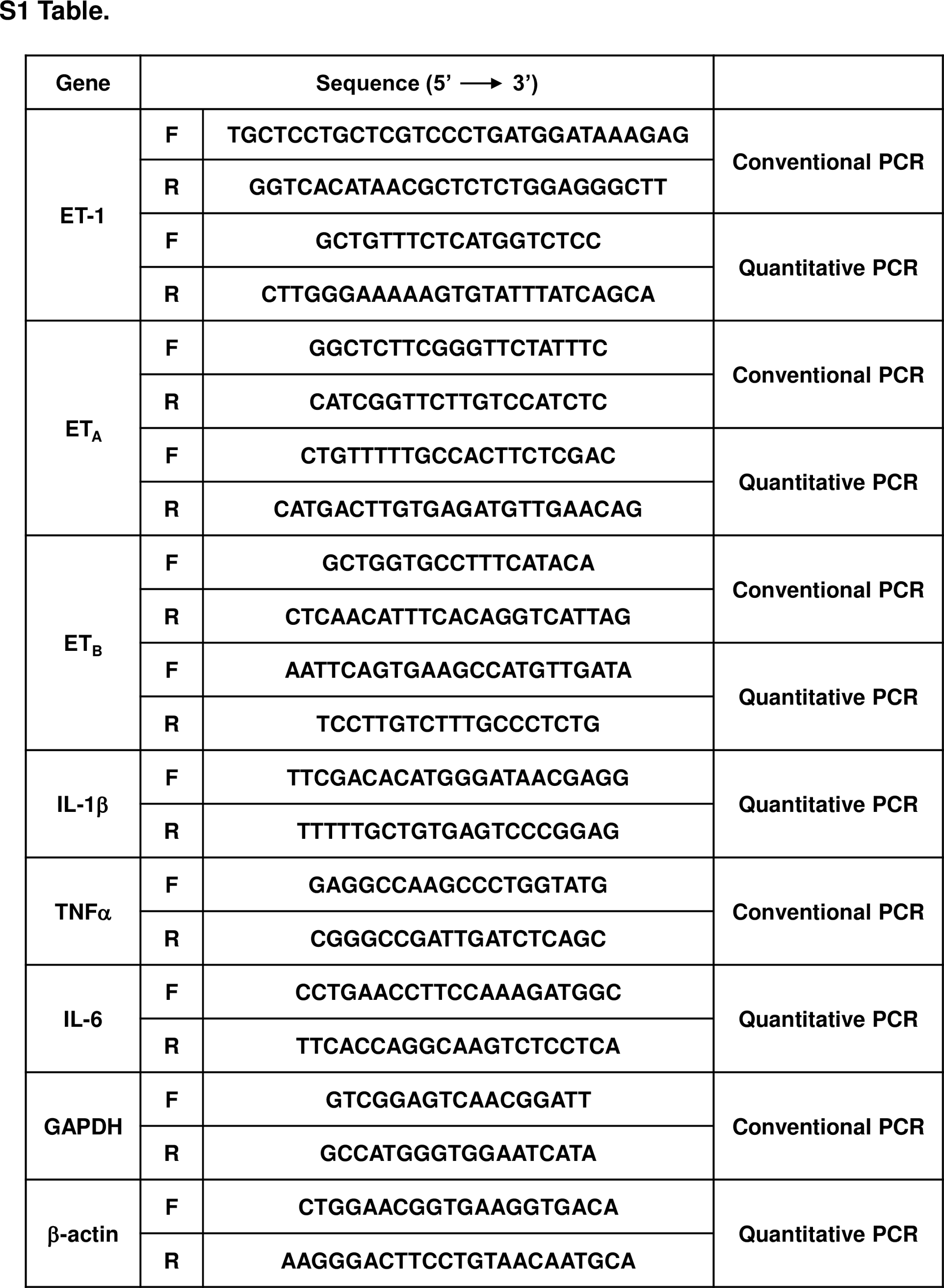

Supplement: S1 Table — (TIF) [file pone.0167713.s001.tif]

## Slide 1
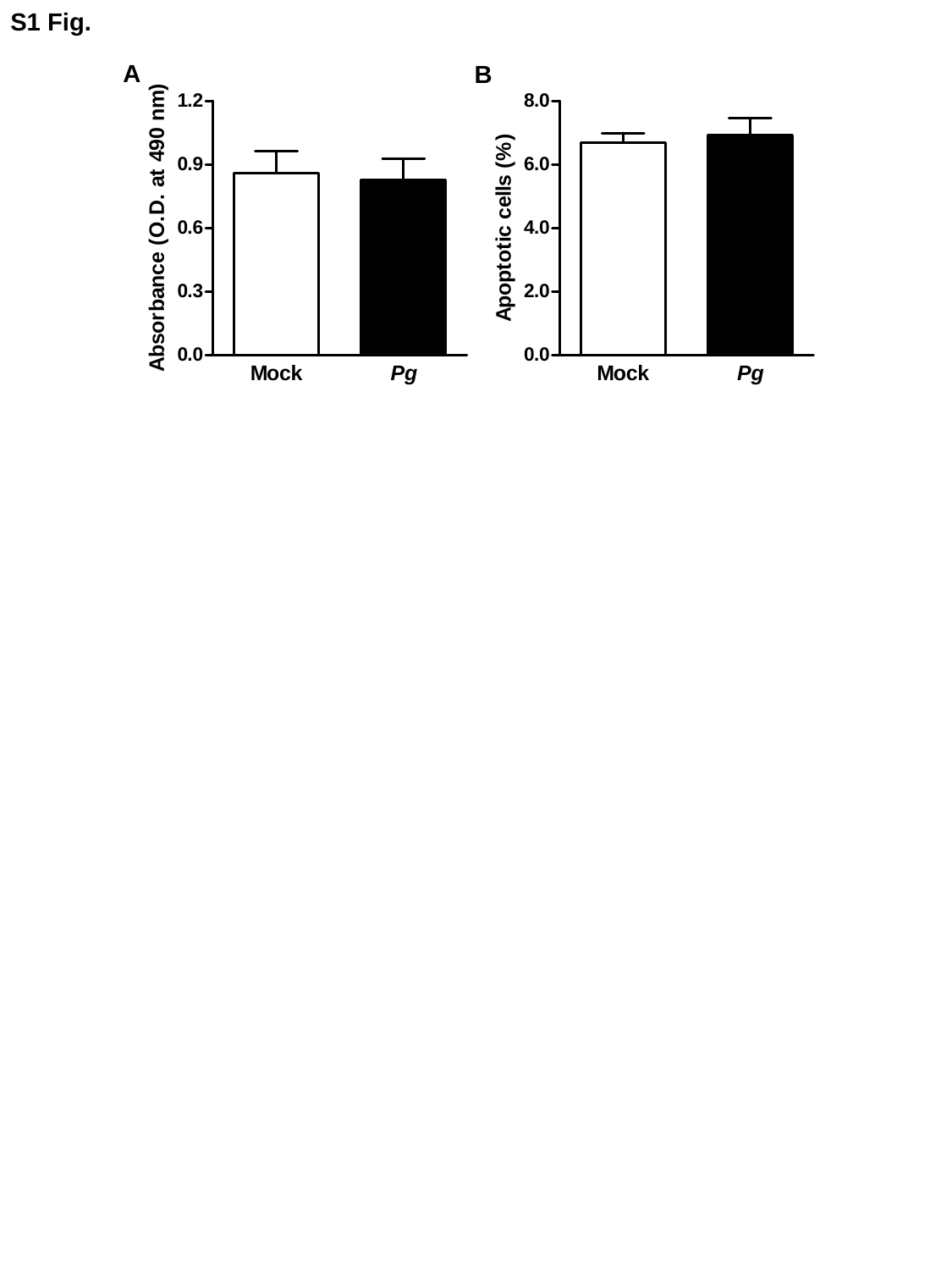

S1 Fig.
A
B

Supplement: S1 Fig — (A and B) After 18h infection with P. gingivalis (100 MOI) apoptotic cell death was determined by flow cytometric analysis using double staining with Annexin V-FITC and 7-AAD (A) and cell growth was examined by MTS assay (B). (PPTX) [file pone.0167713.s002.pptx]

## Slide 1
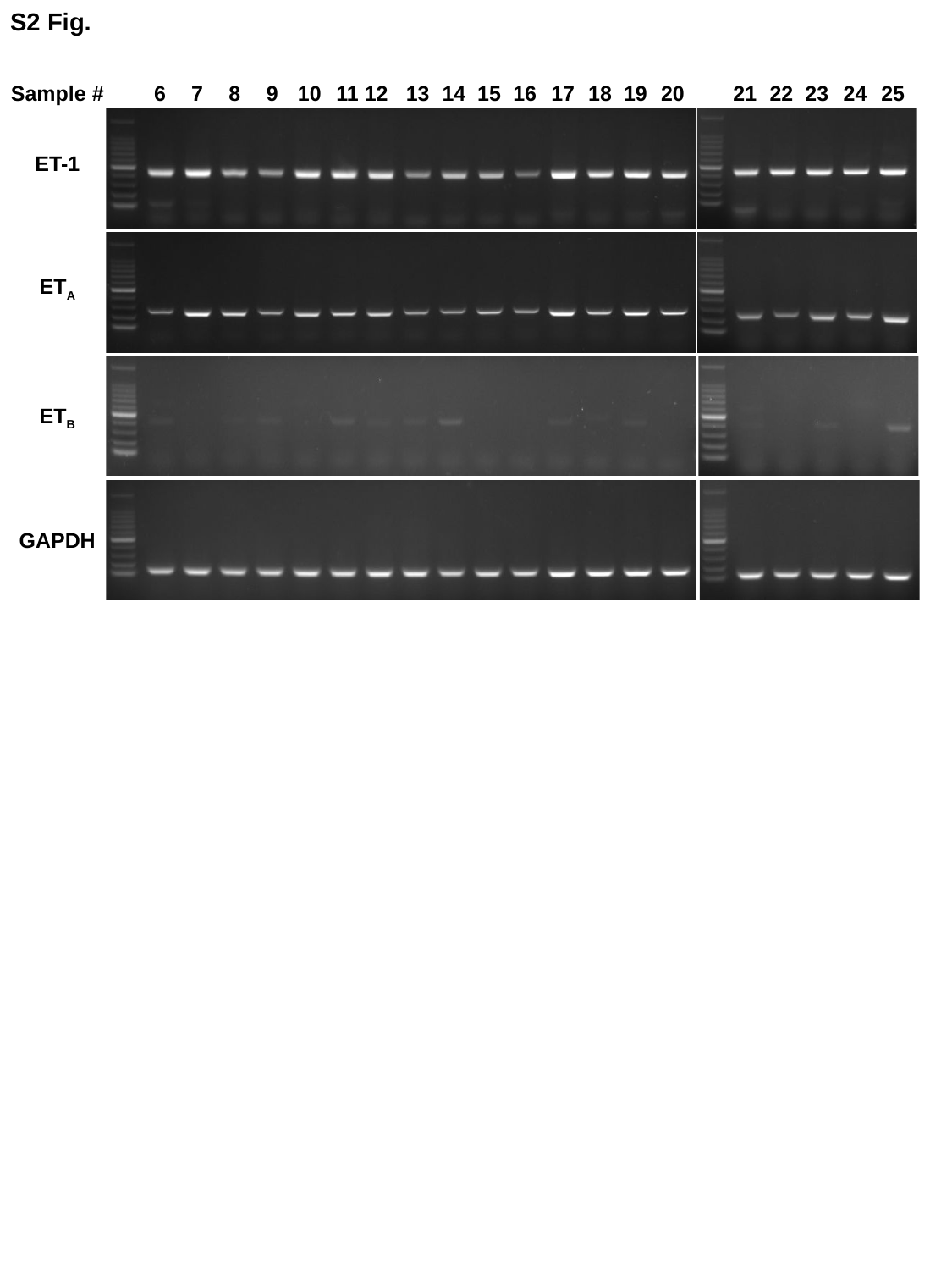

S2 Fig.
Sample #
6
7
8
9
10
11
12
13
14
15
16
17
18
19
20
21
22
23
24
25
ET-1
ETA
ETB
GAPDH

Supplement: S2 Fig — RNA was extracted from human gingival epithelial cells and mRNA expression of ET-1, ETA and ETB in HGECs was analyzed by RT-PCR. GAPDH was used as loading control. (PPTX) [file pone.0167713.s003.pptx]

## Slide 1
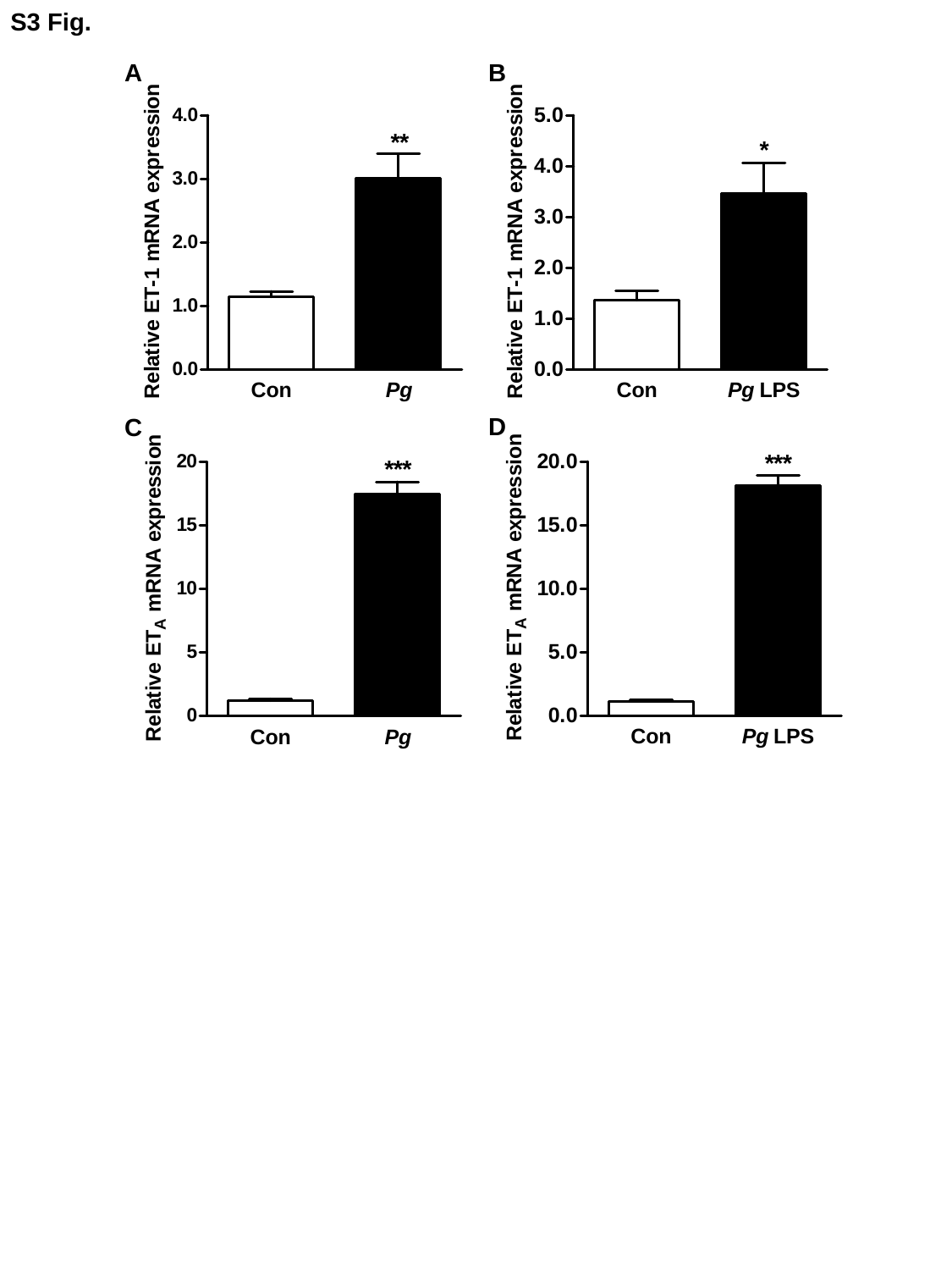

S3 Fig.
A
B
D
C

Supplement: S3 Fig — ET-1 (A and B) or ETA (C and D) mRNA expression was examined by quantitative RT-PCR after Pg (100 MOI; A and C) infection for 18h or Pg LPS (1 μg/ml; B and D) treatment for 24h. *P<0.05; **P<0.01; **P<0.001. (PPTX) [file pone.0167713.s004.pptx]

## Slide 1
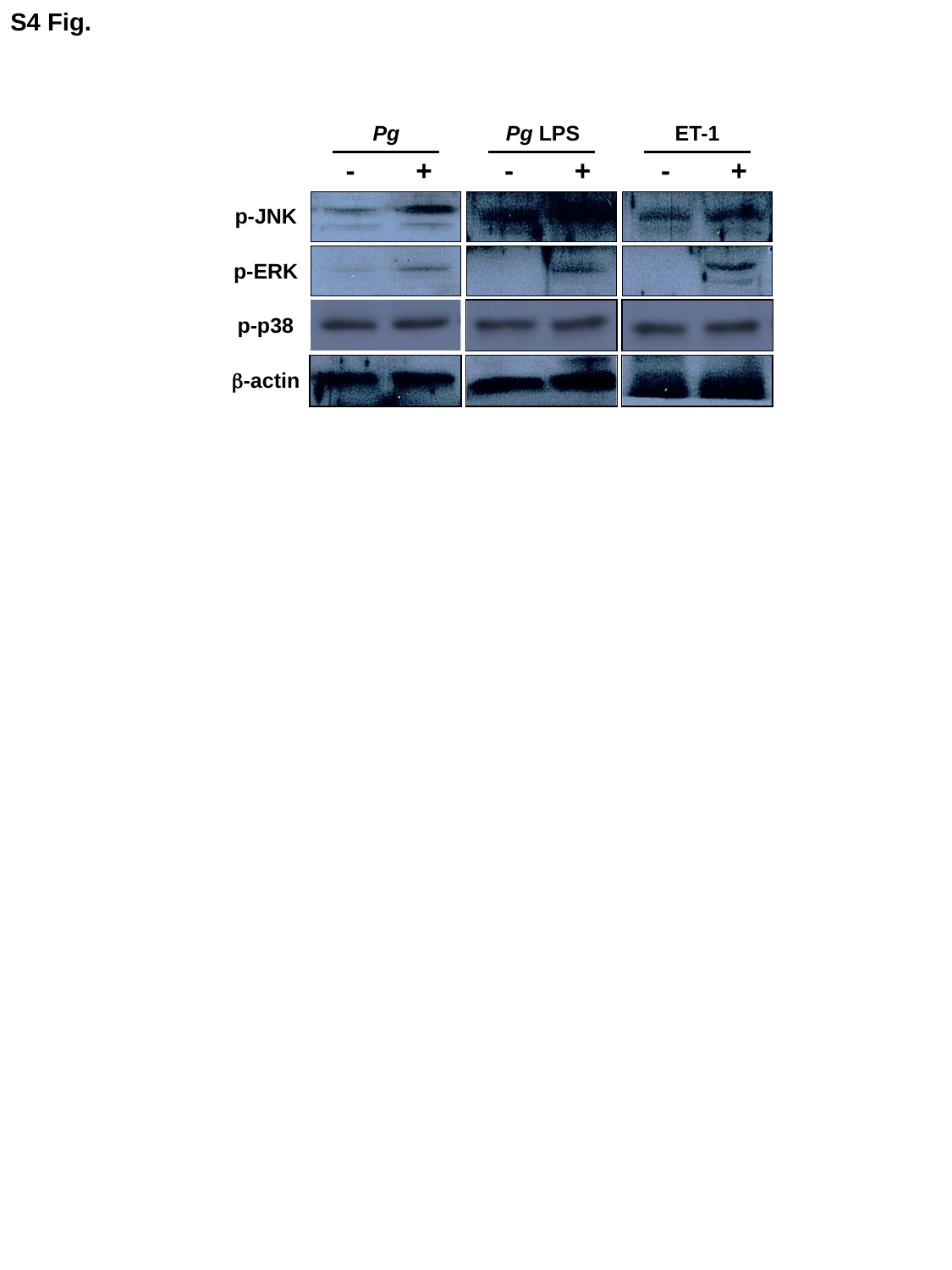

S4 Fig.
Pg
Pg LPS
ET-1
-
+
-
+
-
+
p-JNK
p-ERK
p-p38
b-actin

Supplement: S4 Fig — Phosphorylation of JNK, ERK and p38 was examined by Western blot with phosphorylation-specific antibodies. β-actin was used as a loading control. (PPTX) [file pone.0167713.s005.pptx]

## Slide 1
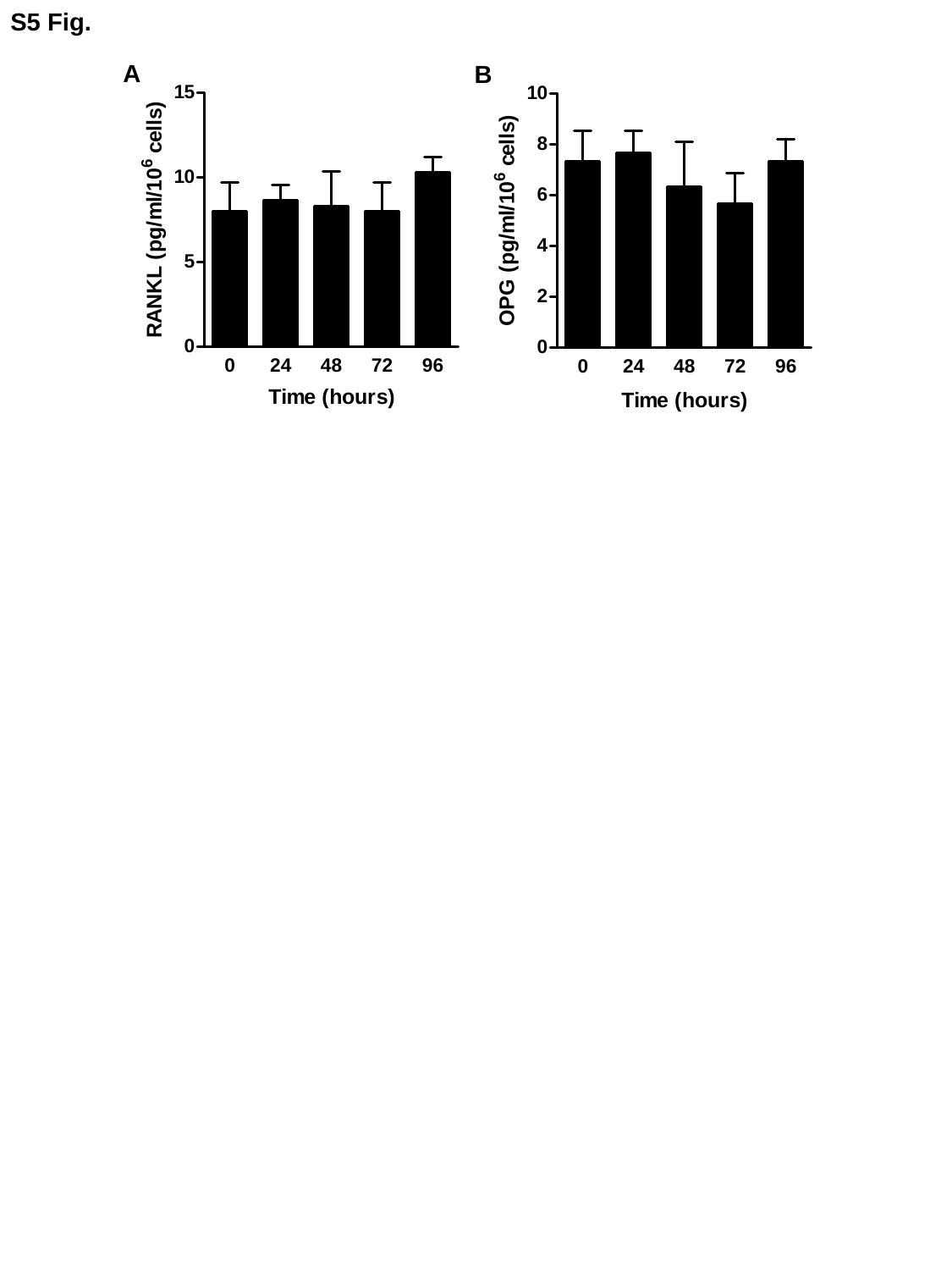

S5 Fig.
A
B

Supplement: S5 Fig — (A and B) ET-1 (100 nM) was treated at the indicated times, and the secretion of RANKL (A) and OPG (B) was examined by ELISA in culture supernatants from five samples (sample #1–5). (PPTX) [file pone.0167713.s006.pptx]
